# Severe neutropenia revealing a rare presentation of dengue fever: a case report

**DOI:** 10.1186/s13104-017-2732-4

**Published:** 2017-08-17

**Authors:** J. Shourick, A. Dinh, M. Matt, J. Salomon, B. Davido

**Affiliations:** Maladies Infectieuses, Hôpital Universitaire Raymond-Poincaré, AP-HP, 104 Bld Raymond Poincaré, 92380 Garches, France

**Keywords:** Antibiotic sparing, Severe neutropenia, Dengue fever, Prolonged neutropenia

## Abstract

**Background:**

Arboviruses are a common cause of fever in the returned traveler often associated with leucopenia, especially lymphopenia and thrombocytopenia. Transient neutropenia has been described in a few cases of arboviruses. However, prolonged and severe neutropenia (<500/mm^3^) has rarely been reported in dengue fever, especially in the returned traveler in Europe.

**Case presentation:**

A 26-year-old healthy female without any medical past history, flying back from Thailand, presented a transient fever with severe neutropenia (<500/mm^3^). Laboratory tests showed a mild hepatic cytolysis and thrombocytopenia, mimicking malaria or viral hepatitis. While she underwent protective isolation, NS1 antigen returned positive in favor of a dengue fever. Outcome was favorable without any antimicrobial therapy.

**Conclusion:**

Physicians should be wary of possible unusual presentation of dengue fever with prolonged neutropenia. Although such biological sign is more often associated with malaria or severe bacterial infection, it may be a sign of arbovirus.

## Background

Arboviruses are a common cause of fever in the returned traveler often associated with leucopenia, especially lymphopenia and thrombocytopenia. Severe neutropenia (defined by <500/mm^3^) in the returned traveler is particularly evocative of malaria whereas typhoid fever causes relative neutropenia. Although it has been firstly reported in France in 1994 [1], neutropenia is a more uncommon sign of dengue fever. Thereafter, cohort studies in Saudi Arabia [2] and more recently in Singapore reported 227 cases (11.8%) [3] of severe neutropenia with a short duration of neutropenia (median of 1 day). Authors showed that severe neutropenia was not associated with an increased risk of superinfection (including nosocomial infection), nor dengue shock syndrome or dengue hemorrhagic fever [3]. In France, a study reported 13 cases of neutropenia (defined by <1500/mm^3^) among 16 cases of dengue fever (81%), without mentioning whether patients had severe neutropenia [4]. They concluded that neutropenia was more often related to dengue fever than other arboviruses such as chikungunya. In an endemic country, distinguishing dengue fever from other infections simply on the basis of clinical and laboratory features has been previously reported [5].

During severe neutropenia in the returned traveler, the absence of infectious complication is widely thought to be due to the really short duration of neutropenia. Unlike hematological aplasia under chemotherapy, it does not systematically require antibiotic prescription. We report the case of a patient being hospitalized in France for a severe neutropenia lasting for more than 72 h due to a dengue fever.

## Case presentation

We report a 26-year-old Caucasian female without any medical past history, apart from being under birth control. She traveled back from Thailand in urban condition for 2 weeks. She was immunized against A and B hepatitis and typhoid fever but did not take any chemoprophylaxis against malaria during her stay. The day she landed in Paris (France) she presented fever, diarrhea, rash and headache. Therefore, she was admitted to Hôpital Raymond-Poincaré, 4 days after the onset of symptoms. She presented fever (38.1 °C) with a skin maculopapular rash with purpura on the wrist and the ankle. Biological tests revealed a severe neutropenia (450/mm^3^) with lymphopenia (540/mm^3^), a thrombocytopenia (100,000/mm^3^) and elevated liver enzymes (2 times above the normal value). Considering she was returning back from a tropical area and had no pre-existing condition, the diagnoses of malaria and typhoid fever have been raised in the first place. She benefited from a malaria smear test, numerous blood cultures and a stool examination to rule out typhoid fever. All microbiological results were negative. As she returned from the South East of Asia and presented of a skin rash with purpuric elements and multiple mosquito bites, physicians hypothesized she could have a dengue fever.

In order to rule out other causes of severe neutropenia, viral PCRs for HIV, EBV, CMV and B19 parvovirus, and serology for C and E hepatitis were performed. The latter returned negative. Moreover, her symptoms were not consistent with a lymphoma considering the absence of organomegaly clinically and on ultrasonography. In addition, lymphocyte immunophenotyping revealed no abnormalities and she had a normal blood cytology. Autoimmune diseases or metabolic deficiencies were also ruled out due to the absence of autoantibodies, a normal level of B12 and B9 vitamins, copper, TSH and blood glucose. Immuno-allergic causes seemed unlikely as she did not take any additional medication.

The day after her admission (D2) her neutrophil count dropped to its lower value (270/mm^3^). Thereafter she underwent protective isolation to avoid superinfection despite remaining afebrile.

Twelve hours after the admission, dengue NS1 antigen returned positive and confirmed the diagnosis of dengue fever. Thereafter PCR confirmed a type 2 dengue fever serotype. Subsequently, the serology returned also positive (IgM+, IgG−) and supported she had a primary dengue infection.

**Fig. 1:**
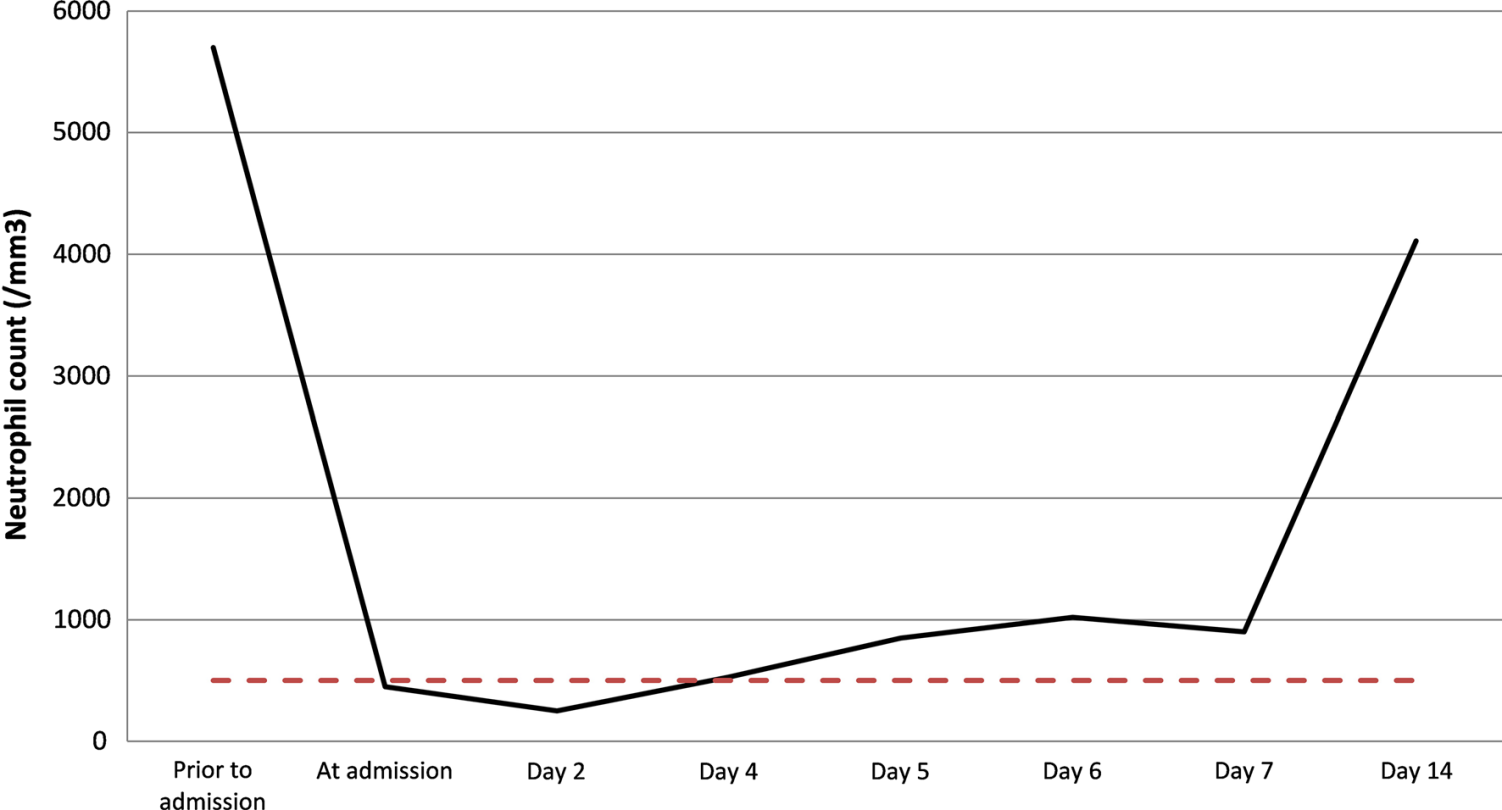
The *black curve* represents the kinetic of neutrophil count over time. The *red dotted-line* stands for the severe neutropenia cut-off value (500/mm^3^)

At D4 her neutrophil count began to increase (530/mm^3^) over the threshold of a severe neutropenia (<500/mm^3^) which authorized to discontinue isolation measures. No antibiotic therapy was initiated as she was clinically stable and in good physical condition. At the follow-up consultation in ambulatory care (D14), she recovered from neutropenia (4110/mm^3^) and was free of symptoms (Fig. 1). Her previous labs attested that her baseline neutrophil count was normal (5700/mm^3^), excluding a potential benign ethnic neutropenia.

## Discussion

In the returned travel from a tropical area, the existence of a neutropenia on the complete blood count is commonly evocative of malaria or typhoid fever [6]. When the latter are suspected, they should be immediately ruled out. However, our case illustrates that arboviruses, in particular dengue fever, can appear as a severe neutropenia in immunocompetent or immunocompromised patients. In the literature dengue fever has also been reported to be a cause of febrile neutropenia in patients suffering from leukemia [7].

Most of the time the decrease of neutrophil count happens within the 24 h after the apyrexia, therefore the clinician can be faced to a non-febrile neutropenia.

Pancharoen et al. reported that primary dengue infection was associated with significantly lower neutrophil count than secondary infection [8]. This may partly explain why our case presented such severe biological signs of neutropenia as she was suffering from a primary dengue infection.

Our case emphasizes that some dengue fever may present as a severe neutropenia lasting for more than 72 h, meanwhile a previous study [3] with a large sample size reported a median duration of 1 day. Nevertheless, authors did not clarify whether some cases may have presented prolonged neutropenia (≥72 h). Such condition requires to closely monitor patient clinically, biologically and radiologically to rule out potential infectious complications or other differential diagnoses.

Interest of GM-CSF has not yet been evaluated for severe neutropenia due to dengue fever. Yet, it is not indicated in short-duration neutropenia, and there is no data supporting a higher risk of superinfection associated with neutropenic dengue fever.

A better knowledge of neutropenic dengue could avoid the use of broad spectrum antibiotics during the first 24 h when patient may still present fever. Promoting such antibiotic sparing of the recommended agents in case of febrile neutropenia [9] (piperacillin/tazobactam or amoxicillin/clavulanate and ciprofloxacin association) is part of the good use of antibiotics.

Considering that dengue incidence is estimated to be 390 million infections (95% credible interval 284–528) per year, of which 96 million (67–136) are symptomatic, there is a true risk that such patients receive inadvertently antibiotics for a neutropenia related to dengue fever. Promoting antibiotic-sparing in an era of continuous development of bacterial resistance [10] is a major health challenge. We believe, the present case must be taken into account in the managing of severe neutropenia in the returned traveler, once malaria and typhoid fever have been ruled out. Also, it must encourage the physician to discontinue their antibiotic prescriptions when there is no relevant data in favor of a bacterial infection.

## Conclusion

While neutropenia is commonly encountered in arboviruses’ endemic countries, such biological sign is more often associated with malaria or severe bacterial infection. Yet, severe neutropenia may still be a sign of authentic arbovirus. Therefore, physicians should be wary of possible unusual presentation with prolonged neutropenia.

## Abbreviations

PCR: polymerase chain-reaction
HIV: human immunodeficiency virus, Ebstein barr virus, cytomegalovirus
CRP: C-reactive protein
ICU: Intensive Care Unit
HIV: human immunodeficiency virus
TSH: thyroid stimulating hormon
GM-CSF: granulocyte macrophage colony stimulating factor

## References

[1] Blanche P, Bredoux H, Abad S, Dreyfus F, Sicard D. Severe neutropenia in dengue. Presse Med. 1994;23:1224. 7831220

[2] Khan NA, Azhar EI, El-Fiky S, Madani HH, Abuljadial MA, Ashshi AM (2008). Clinical profile and outcome of hospitalized patients during first outbreak of dengue in Makkah, Saudi Arabia. Acta Trop..

[3] Thein T-L, Lye DC, Leo Y-S, Wong JGX, Hao Y, Wilder-Smith A (2014). Severe neutropenia in dengue patients: prevalence and significance. Am J Trop Med Hyg..

[4] Hochedez P, Canestri A, Guihot A, Brichler S, Bricaire F, Caumes E (2008). Management of travelers with fever and exanthema, notably dengue and chikungunya infections. Am J Trop Med Hyg..

[5] Chadwick D, Arch B, Wilder-Smith A, Paton N (2006). Distinguishing dengue fever from other infections on the basis of simple clinical and laboratory features: application of logistic regression analysis. J Clin Virol.

[6] Cooper EC, Ratnam I, Mohebbi M, Leder K (2014). Laboratory features of common causes of fever in returned travelers. J Travel Med..

[7] Jain H, Sengar M, Menon H, Dangi U, Biswas S, Chandrakanth MV (2014). Dengue fever as a cause of febrile neutropenia in adult acute lymphoblastic leukemia: a single center experience. Hematol Oncol Stem Cell Ther.

[8] Pancharoen C, Mekmullica J, Thisyakorn U (2001). Primary dengue infection: what are the clinical distinctions from secondary infection?. Southeast Asian J Trop Med Public Health..

[9] Freifeld AG, Bow EJ, Sepkowitz KA, Boeckh MJ, Ito JI, Mullen CA (2011). Clinical practice guideline for the use of antimicrobial agents in neutropenic patients with cancer: 2010 update by the infectious diseases society of America. Clin Infect Dis..

[10] Carlet J, Pulcini C, Piddock LJV (2014). Antibiotic resistance: a geopolitical issue. Clin Microbiol Infect..

